# 3BDO inhibits the proliferation, epithelial-mesenchymal transition (EMT), and stemness via suppressing survivin in human glioblastoma cells

**DOI:** 10.7150/jca.66674

**Published:** 2022-01-16

**Authors:** Zhaotao Wang, Yongping Li, Minyi Liu, Danmin Chen, Jiajie Lu, Yunxiang Ji, Zhou Xing, Yezhong Wang

**Affiliations:** Institute of Neuroscience, Department of Neurosurgery, the Second Affiliated Hospital of Guangzhou Medical University, Guangzhou 510260 China

**Keywords:** 3BDO, glioblastoma, EMT, GSC, survivin

## Abstract

**Background:** Glioblastoma (GBM) is a tumor of the central nervous system with an extremely poor prognosis. Stemness and EMT play important roles in GBM progression. 3-benzyl-5-((2-nitrophenoxy) methyl) dihydrofuran-2(3H)-one (3BDO), an autophagy inhibitor, has been reported to exert anti-cancer activities on lung carcinoma. However, the effects of 3BDO on GBM remain unknown. Therefore, the purpose of this study was to explore the effects of 3BDO on GBM and to investigate the underlying molecular mechanisms.

**Method:** CCK-8 experiments and clone formation assays were conducted to determine the level of cell proliferation. Transwell assay was conducted to examine cell migration and invasion abilities. Western blotting and immunofluorescence staining were used to analyze protein expression levels. A xenograft mouse model was used to evaluate the effect of 3BDO *in vivo*.

**Results:** We found that 3BDO inhibited U87 and U251 cell proliferation in a dose-dependent manner. Additionally, 3BDO decreased the degree of sphere formation and levels of stemness markers (sox2, nestin, and CD133) in GSCs. 3BDO also inhibited migration and invasion abilities and suppressed EMT markers (N-cadherin, vimentin, and snail) in GBM cells. Moreover, we found that 3BDO downregulated the expression of survivin in both GBM cells (U87, U251) and GSCs. Furthermore, overexpression of survivin decreased the therapeutic effect of 3BDO on EMT, invasion, migration, and proliferation of GBM cells, as well as decreased the stemness of GSCs. Finally, we demonstrated that 3BDO could inhibit tumor growth in a tumor xenograft mouse model constructed using U87 cells. Similar to the *in vitro* findings, 3BDO decreased the expression of survivin, EMT makers, and the degree of stemness *in vivo*.

**Conclusions:** Our results demonstrate that 3BDO can repress GBM both *in vitro* and *in vivo* via downregulating survivin-mediated stemness and EMT.

## Background

Glioblastoma (GBM) is a high-grade glioma with high rates of invasion and raid growth. Despite the development of surgical and medical treatment modalities, patients diagnosed with GBM rarely survive longer than 15 months after diagnosis 1, 2. Currently, temozolomide (TMZ) is the primary first-line agent used in GBM chemotherapy, but its effectiveness has been marred by drug resistance. Therefore, other agents with higher efficacy need to be urgently identified to manage GBM.

Cancer recurrence and metastasis are often attributed to cancer stem-like cells (CSCs), given their capabilities in regenerating tumors by resisting standard chemotherapy and radiotherapy regimens 3-5. A myriad of cancers has been shown to harbor CSCs, including GBM. CSCs in GBM are known as glioma stem cells (GSCs) and have been shown to be able to undergo multi-lineage differentiation, self-renewal, and extensive proliferation. Current research has suggested that these GSCs function as pivotal molecules for GBM growth, with their elimination leading to potential GBM growth inhibition 6-8. It is therefore of great interest to explore the potential use of GSCs as a means of GBM treatment.

The epithelial-mesenchymal transition (EMT) is the mechanism by which normal epithelial cells acquire a mesenchymal phenotype. Cell EMT is the foundation of cancer development, and this process increases the motility of individual cancer cells and allows them to invade across epithelial junctions and extracellular matrices 9, 10. Therefore, the importance of EMT in GBM is strongly supported. Both *in vivo* and *in vitro* studies on GBM have shown that the activation of an EMT-like program results in enhanced malignant cell migration and invasion 11, 12. Additionally, patients with aggressive GBM and a poor prognosis invariably demonstrate elevated levels of mesenchymal markers, such as N-cadherin, snail, and vimentin 13, 14. Therefore, EMT inhibition appears to be a feasible modality of treating GBM.

Survivin is a protein associated with the inhibition of apoptosis and is also known as a baculoviral inhibitor of apoptosis repeat-containing 5 (BIRC5). BIRC5 levels have been found to be significantly elevated in multiple cancers, such as colon cancer, lung cancer, breast cancer, and melanoma 15, 16. Similarly, an increasing number of studies have found significantly elevated survivin expression levels in glioma cells, compared with normal central nervous tissue, along with a positive association between survivin expression and pathological glioma grade. Patients with higher levels of survivin have been shown to have a worse prognosis 17. In addition, increased survivin expression promoted EMT and the stemness process 16, 18.

3-Benzyl-5-((2-nitrophenoxy) methyl)-dihydrofuran-2 (3H)-one (3BDO) is an mTOR activator that functions as an autophagy inhibitor. For example, it has been reported that 3BDO exposure significantly diminishes the number of autophagosomes in APP/PS1 transgenic mice while simultaneously improving memory function 19. In addition, 3BDO partially reversed the maturation of Rheb1-deficient neutrophils through mTOR inactivation 20. Furthermore, accumulating evidence has demonstrated that 3BDO may act independently as an mTOR modulator. For instance, 3BDO alleviated plaque endothelial cell death and slowed down the establishment of atherosclerosis in mice, but in a manner that was not dependent on autophagy or mTOR activity 21. 3BDO has also been demonstrated to function as an inflammatory suppressor both *in vivo* and *in vitro* 22. Furthermore, 3BDO has also been shown to inhibit cancer cell growth when administered in combination with DPB 23. However, it is not well known if 3BDO exerts potential anti-cancer properties in GBM.

This study first dissected the impact of 3BDO on human GBM *in vitro*. Then, experiments that specifically evaluated EMT and stemness-related properties and their association survivin expression were conducted. The effect of 3BDO on tumor xenograft mice models was also investigated, and it was found to be able to inhibit tumor growth. This study not only identified 3BDO as a potential agent but also provides a theoretical foundation for a novel approach to GBM treatment.

## Materials and Methods

### Chemicals, Reagents, and Antibodies

3BDO was obtained from Selleck. DMSO was used to produce a 3BDO suspension which was kept at 4 °C. Dulbecco's modified Eagle's medium (DMEM) and fetal bovine serum (FBS) were procured from (Grand Island, USA). Antibodies against N-cadherin (#13116T), nestin (#33475S), CD133 (#64326S), vimentin (#5741T), GAPDH (#5174) and survivin (#2802S) were bought from Cell Signaling Technology (Beverly, MA). Abcam (Cambridge, MA) provided antibodies against snail (ab180714) and sox2(ab97959).

### Cell Culture

The glioblastoma cell lines, U87 and U251, were obtained from the Chinese Academy of Medical Sciences (Beijing, China). All cell lines were maintained in DMEM supplemented with 10% FBS. GSC is a kindly gift from Jia Ouyang from SooChow university, then cultured in GSC medium. Briefly, fresh tumor samples were dissociated into single cells using Accutase (Gibco), filtered and resuspended in GCS medium (Neurobasal, 1× B27, 20 ng/ml EGF, 20 ng/ml bFGF, and 1×GlutaMax). Briefly, GSC populations were isolated by fluorescence-activated cell sorting (FACS) after 6-18 hours recovery in GSC medium. FcR blocking reagent, CD133/2 (293C3)-VioBright FITC and isotype control IgG2b-VioBright FITC antibodies (Miltenyi) were used according to the instructions. The GSCs were cultured in GSC medium, which is a neurobasal medium containing 20 ng/ml basic fibroblast growth factor (bFGF) (Peprotech, USA), 2% B27 (Gibco, USA), 1% Glutamine (Gibco, USA), and 20 ng/ml epidermal growth factor (EGF) (Peprotech, USA). All the above mentioned cells were incubated in a humidified atmosphere with 5% CO_2_ at 37 °C.

### Cell Viability Assay

Cells were digested and suspended in a 96-well plate at a density of 4 × 10^3^ cells/well for 24 h. Each well was exposed to various concentrations of 3BDO. After 24 h, the cells were further treated with 10 µl of CCK-8 solution and allowed to incubate for an hour at 37°C. A microplate reader was used to determine cell viability.

### Clonogenic Assay

Cells were suspended in DMEM medium containing 10% FBS. 500 cells were added to a plate with a diameter of 6 cm. The selected 3BDO concentrations were added, and the cells were cultured for 1 week at 37°C in a 5% CO_2_ atmosphere until cell colonies were visible. The cells were then rinsed thrice with PBS before fixation with methyl alcohol and stained with crystal violet for 10 min. The number of colonies was counted after three final rinses with PBS. The experiments were performed in triplicate.

### Sphere Formation Assay

Single GSC cells (3000 cells per well) were plated onto a 6 cm ultra-low attachment plate (Corning) used above containing GSC medium, following 3BDO treatment, as indicated. Cells were cultured under standard cell culture conditions. After 5 days of culture, the number of spheres larger than 50 μm was counted under a microscope. The experiment was performed in triplicates, and at least 5 fields of view were evaluated in each replicate.

### Cell migration and Invasion Assay

A Transwell system (Corning, USA) was used to study cell invasion and migration abilities. The upper chamber with 8 µm pores was used to house the cells (2 × 10^4^ suspended 200 µl of DMEM supplemented with 1% FBS). The chambers were coated with 100 µl of matrigel (B.D. Biosciences, CA, USA) to conduct the migration assays or left uncoated for the invasion assays. 600 µl of DMEM supplemented with 20% FBS was placed in the lower chamber along with various concentrations of 3BDO. The cells were incubated for 24 hours. After this period, the cells were removed from the upper chamber using a cotton swab, and cells in the lower chamber were fixed with ethanol and stained with crystal violet. The number of cells was counted in three random fields viewed at 100x magnification.

### Transfection

Both GSCs and the GBM cell lines were transfected with a survivin gene-containing plasmid to produce cells that overexpressed survivin (GeneCopoeia, Maryland Rockville, USA) using jetPRIME® Versatile DNA transfection reagent (Polyplus Transfection, France). Control cells were transfected with the vector only. All experiments were conducted as instructed by the manufacturer.

### Western Blotting Assays

Xenograft glioblastoma tissue homogenates and cell lysates were used for the western blotting assays. The Pro-prep TM protein Extraction Solution (iNtRON Biotechnology, Korea) was used to extract proteins. 10-12% sodium dodecyl sulfate-polyacrylamide gel electrophoresis (SDS-PAGE) was performed to separate the component proteins before blotting them onto polyvinylidene difluoride membranes (Merck, KGaA, Darmstadt, Germany). 5% BSA was used to block unspecific reactions for an hour at room temperature. The membranes were then incubated overnight with specific primary antibodies at 4°C. Horseradish protein-conjugated secondary antibodies were exposed to the membranes for an hour at room temperature. A Super Signal ECL (Pierce, Rockford, IL, USA) system was used to interpret the results. Samples derived from the same experiment and their gels/blots were parallel processed.

### Immunofluorescence

After the treatments indicated above were conducted, the GSCs were incubated on cell climbing slices coated with poly-lysine for 1 hour. This was followed by immunofluorescence staining in accordance with standard protocols. 1× PBS was first used to rinse the slides thrice before normal goat serum supplemented with 0.3% Triton X-100 was added and allowed to incubate for an hour at room temperature. Then, specific primary antibodies suspended in 300 μl of 5% BSA were added onto the slides and allowed to incubate overnight at 4°C. The cells were once again washed thrice with PBS the next morning and exposed to Alexa Fluor 555-conjugated secondary antibody at room temperature for 45 minutes. Then, the slides were once again rinsed thrice and counterstained with DAPI for 10 min, washed, and air-dried. Images were captured under either light or fluorescence microscopy under a confocal microscope.

### U87 Xenograft Mouse Model with 3BDO Treatment

Female BALB/c nude mice were procured from the Animal Experiment Center of Southern Medical University (Guangzhou, China). The mice were 6-8 weeks old and were reared under institutional animal care guidelines after the experimental procedures involving animals were approved by the Institutional Animal Care Committee of the Institutional Animal Care of the Second Affiliated Hospital of Guangzhou Medical University. Guidelines for the ethical review of laboratory animal welfare (People's Republic of China National Standard GB/T 35892) and ARRIVE guidelines (https://www.nc3rs.org.uk/arrive-guideline) were also followed 24. All mice received subcutaneous injections containing cultured U87 cells (5 × 10^6^ cells per mouse) to the dorsum. Calipers were used to assess tumor size in two orthogonal directions. Tumor volume (mm^3^) was derived based on the following formula: 1/2 × length × width^2^. Upon achieving a tumor size of approximately 150 mm^3^, the mice were either intraperitoneally injected with the vehicle or 3BDO (80 mg/kg/day) (n=5 mice per group). The body weight and tumor size of the mice were assessed once every 5 days. All tumors were harvested for further analysis after the mice were sacrificed at the end of these experiments.

### Statistical analysis

All data indicated are the average of the results of experiments performed in triplicate and were analyzed using SPSS 20.0 software. An independent T-test was used to perform simple comparisons between 2 groups, and while comparisons between multiple groups were evaluated using one-way analysis of variance, followed by post hoc analysis, using Dunnett's T3 test or Turkey test. A P < 0.05 was interpreted as a statistically significant result.

## Results

### 3BDO inhibits the proliferation, migration, and invasion of GBM cells

**Figure 1:**
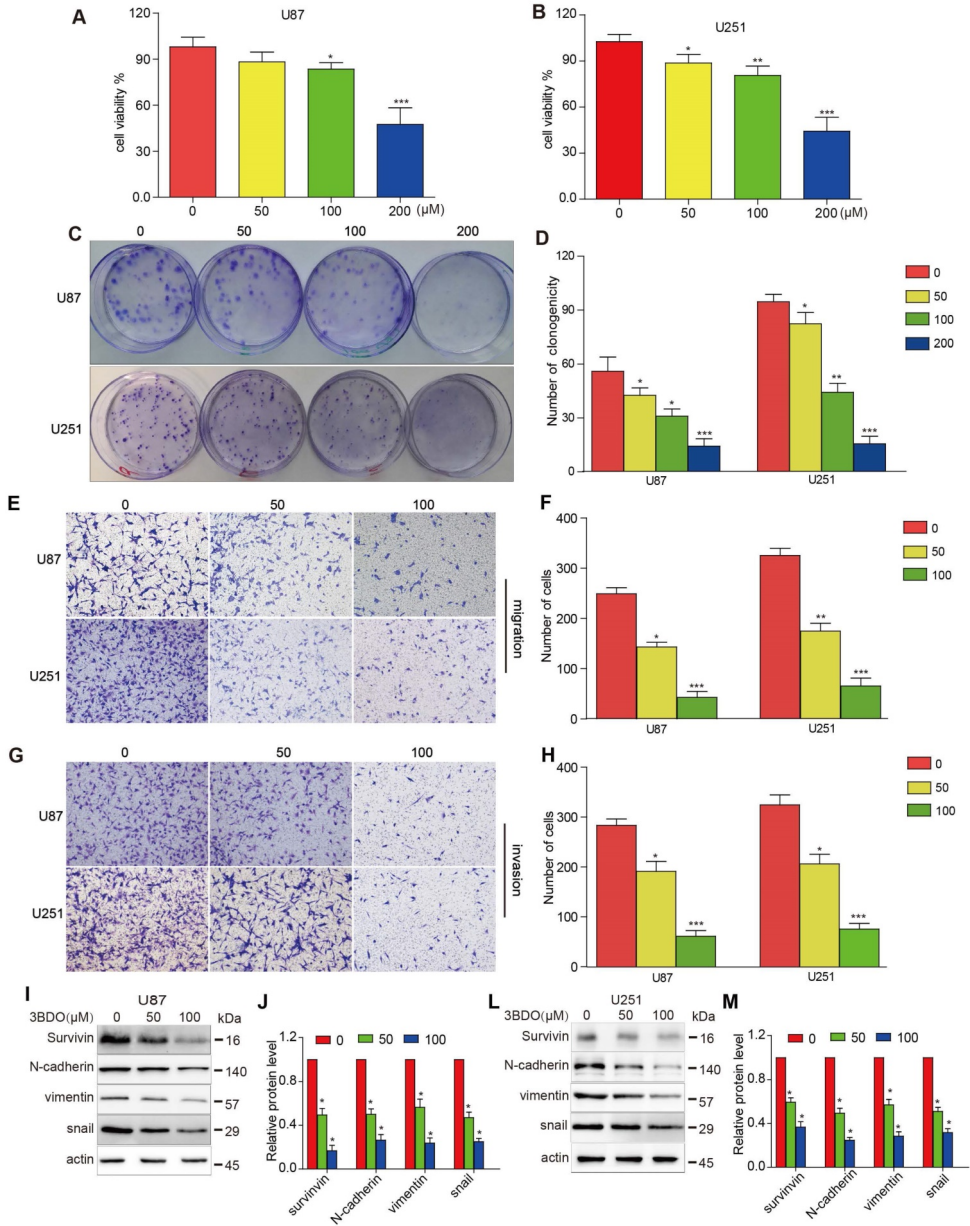
** 3BDO diminishes the invasion, migration, and proliferation abilities and the expression of EMT-associated molecules and survivin in GBM cells.** (A-B) U87 and U251 cells were treated with 3BDO for 24 h, followed by tests to confirm cell viability. (C-D) U87 and U251 cells were treated with 3BDO for 7 days before the clonogenic assay was performed. (E-H) GBM cells were incubated with selected 3BDO concentrations for 24 hours and were subjected to a transwell assay to evaluate the degree of cell invasion and migration. (I-M) Western blotting analysis was used to investigate the expression of survivin, and EMT-associated proteins in U87 and U251 cells exposed to selected concentrations of 3BDO for 24 h, and the gels were analyzed. n=3 or n=4 in each group. All experiments were performed in triplicate. *, P < 0.05 compared with the control (0 μM).

As shown in Figure 1 A-B, U87 and U251 cell growth were inhibited by 3BDO in a dose-dependent manner after 24 hours of treatment. Similarly, 3BDO also suppressed the colony formation ability of the cells in a dose-dependent manner (Figure 1C-D). Next, we observed the invasive and migratory abilities of cells treated with 3BDO using transwell assays. In contrast to the untreated groups, the 3BDO treated groups exhibited lower rates of cell invasion and migration in a dose-dependent manner (Figure 1E-H). These *in vitro* findings strongly suggest that 3BDO inhibits the proliferation, invasion, and migration of GBM cells.

### 3BDO decreases EMT marker expression and inhibits survivin expression in GBM cells

Exposure to 50 μM or 100 μM doses of 3BDO for 24 hours resulted in a lower level of N-cadherin, vimentin, and snail expression than the 0 μM group (Figure 1I-M). Meanwhile, 3BDO reduced survivin expression levels in a dose-dependent manner in U87 and U251 cells. Our findings indicate that 3BDO decreases the expression of EMT markers and inhibits survivin expression in GBM cells.

### 3BDO inhibits cell growth and downregulates survivin expression and stemness markers in GSCs

**Figure 2:**
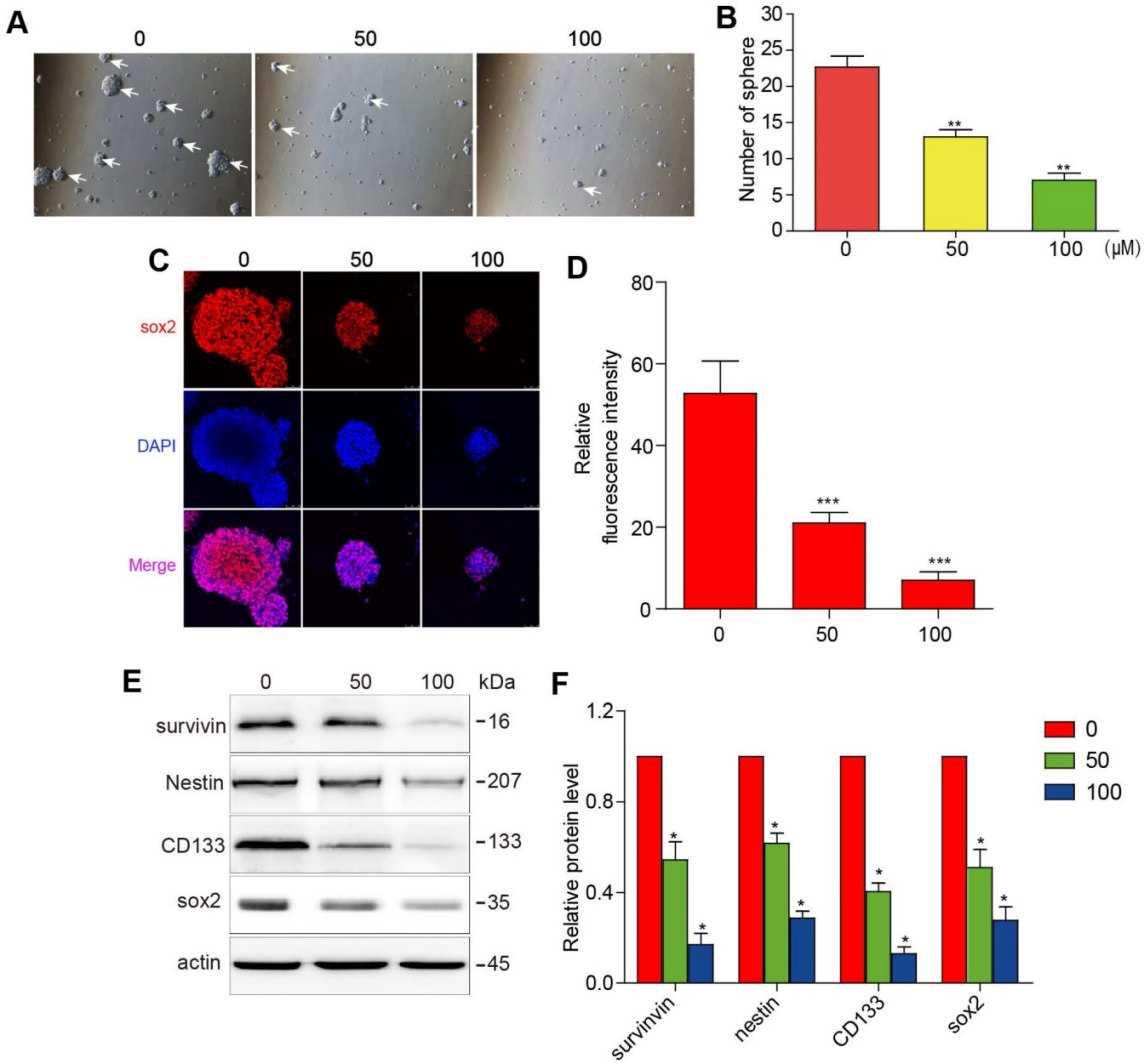
** 3BDO inhibits cell growth and downregulates the expression of survivin and stemness markers in GSCs.** GSCs were cultured with selected doses of 3BDO for 72 h and were then subjected to (A-B) quantification of the spheres formed, (C-D) immunofluorescence examination to determine sox2 expression levels, (E-F) western blotting analysis to determine protein expression levels of stemness markers and the gels were analyzed. The GSC sphere (White arrow). n=3 or n=4 in each group. All experiments were performed in triplicate. *, P < 0.05; **, P < 0.01; ***, P < 0.001, compared with the control (0 μM).

The initiation and progression of GBM have been attributed to the effect of GSCs. It is, therefore, of sound scientific reasoning to pursue GSC as a therapeutic target in GBM. The GSCs were cultured in a sphere-forming medium and were treated with different concentrations of 3BDO for 72 h. As shown in figure 2A-B, 3BDO treatment decreased the number of GSC spheres in a dose-dependent manner.

Sox2 is an important marker of GSC stemness. We found that sox2 expression levels decreased as 3BDO concentration increased (Figure 2C-D). Next, western blotting analysis was performed to quantify the expression of stemness markers, such as nestin, CD133, and sox2. The results showed that the aforementioned stemness marker expression levels were downregulated after incubation for 24 h with the indicated doses of 3BDO. Moreover, we found that 3BDO decreased survivin expression levels in a dose-dependent manner in GSCs (Figure 2E-F). These results suggest that 3BDO is able to suppress stemness and downregulate survivin levels in GSCs.

### Overexpression of survivin decreased the effect of 3BDO on GBM cells and GSCs

**Figure 3:**
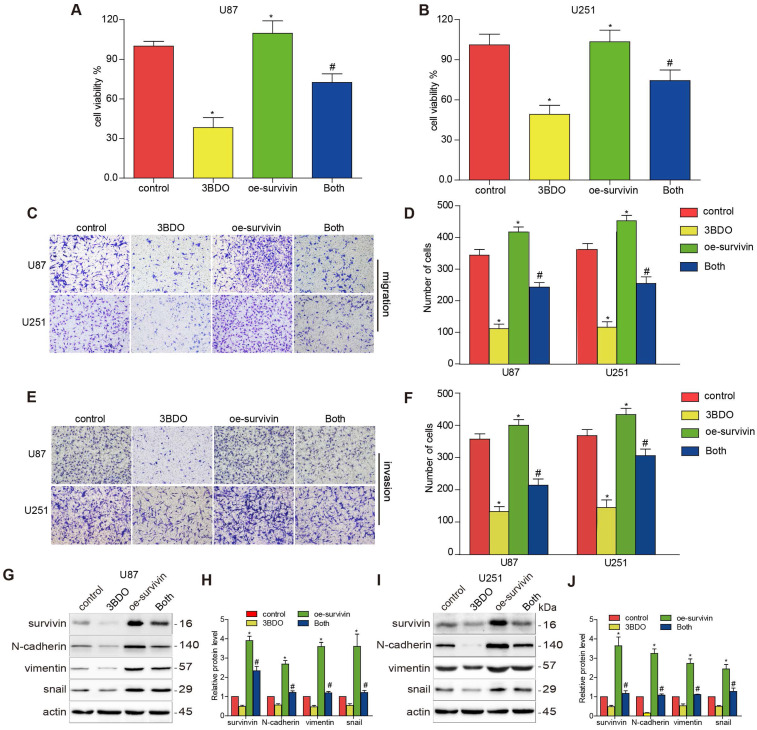
**Overexpression of survivin decreases the inhibition effect on cell proliferation, migration, invasion, and EMT induced by 3BDO.** (A-B) GBM cells were transfected with survivin or the vector plasmid for 12 hours before being transferred onto 96-well plates and were treated with 100 μM 3BDO or the vehicle for 24 hours. Cellular proliferation was evaluated using a CCK-8 assay. (C-F) Migration and invasion assays were performed on transfected GBM cells using a Transwell assay. (G-J) GBM cells transfected with survivin, or the vector plasmid were treated with 100 μM 3BDO for 24 h. Western blotting analysis was performed to detect protein expression levels, and the gels were analyzed. *, P < 0.05 vs. control; ^#,^ P < 0.05, compared with either 3BDO incubation or survivin transfection alone.

Previous studies have highlighted the role of survivin in EMT. We aimed to confirm whether 3BDO-induced EMT suppression was mediated by survivin inhibition. U87 and U251 cells were artificially induced to overexpress survivin before being subjected to a 24 h of incubation with either 3BDO or the vehicle. The proliferation, migration, and invasion abilities of the GBM cells increased in the presence of survivin overexpression (Figure 3A-F). We also found that survivin upregulation increased the expression of EMT markers, including N-cadherin, vimentin, and snail (Figure 3G-J). Furthermore, we found that the overexpression of survivin reduced the inhibitory effects of 3BDO on GBM cells.

**Figure 4:**
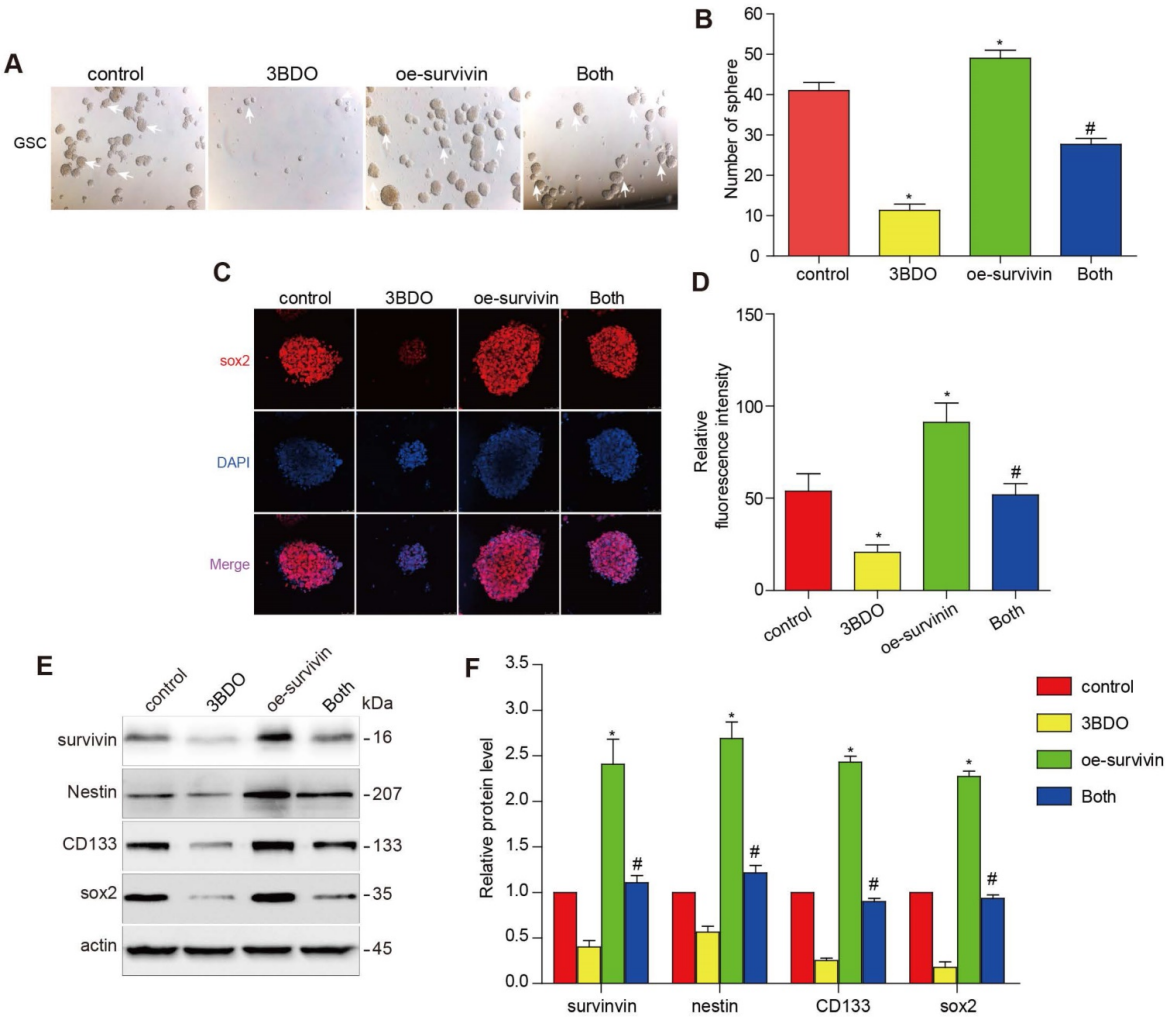
** Overexpression of survivin decreases the impact of 3BDO on GSCs.** GSCs were transfected with survivin or the vector plasmid for 12 hours and then cultured with the 100 μM 3BDO. Additionally, the GSCs were incubated with or without 100 μM 3BDO for 72 h and then subjected to, (A-B) quantification of the spheres formed (C-D) immunofluorescence examination to determine sox2 expression levels, (E-F) western blotting analysis to determine protein expression levels of the stemness markers and the gels were cropping. The GSC sphere (White arrow). n=3 or n=4 in each group. All experiments were performed in triplicate. *, P < 0.05; **, P < 0.01; ***, P < 0.001, compared with the control (0 μM).

Multiple studies have reported that survivin is abundantly expressed abundantly in GBM tissues and also in human-derived GSC cultures. The downregulation of survivin is abolished both *in vivo* and *in vitro* during GSC growth. To further investigate whether 3BDO inhibited GSCs through its effect on survivin, we translocated the cells with a survivin overexpressing plasmid or its vector plasmid and subsequently incubated the cells with 3BDO. We found that the overexpression of survivin enhanced the number of spheres formed and upregulated stemness markers, such as nestin, CD133, and sox2. Moreover, the overexpression of survivin reduced the effect of 3BDO on sphere formation and the upregulation of stemness markers in the GSCs (Figure 4). These results suggest that 3BDO exerts an anti-tumor effect by suppressing survivin-triggered EMT in GBM cells and by exerting an anti-stemness effect on GSCs.

### 3BDO suppressed GBM growth in a U87 xenograft mouse model

**Figure 5:**
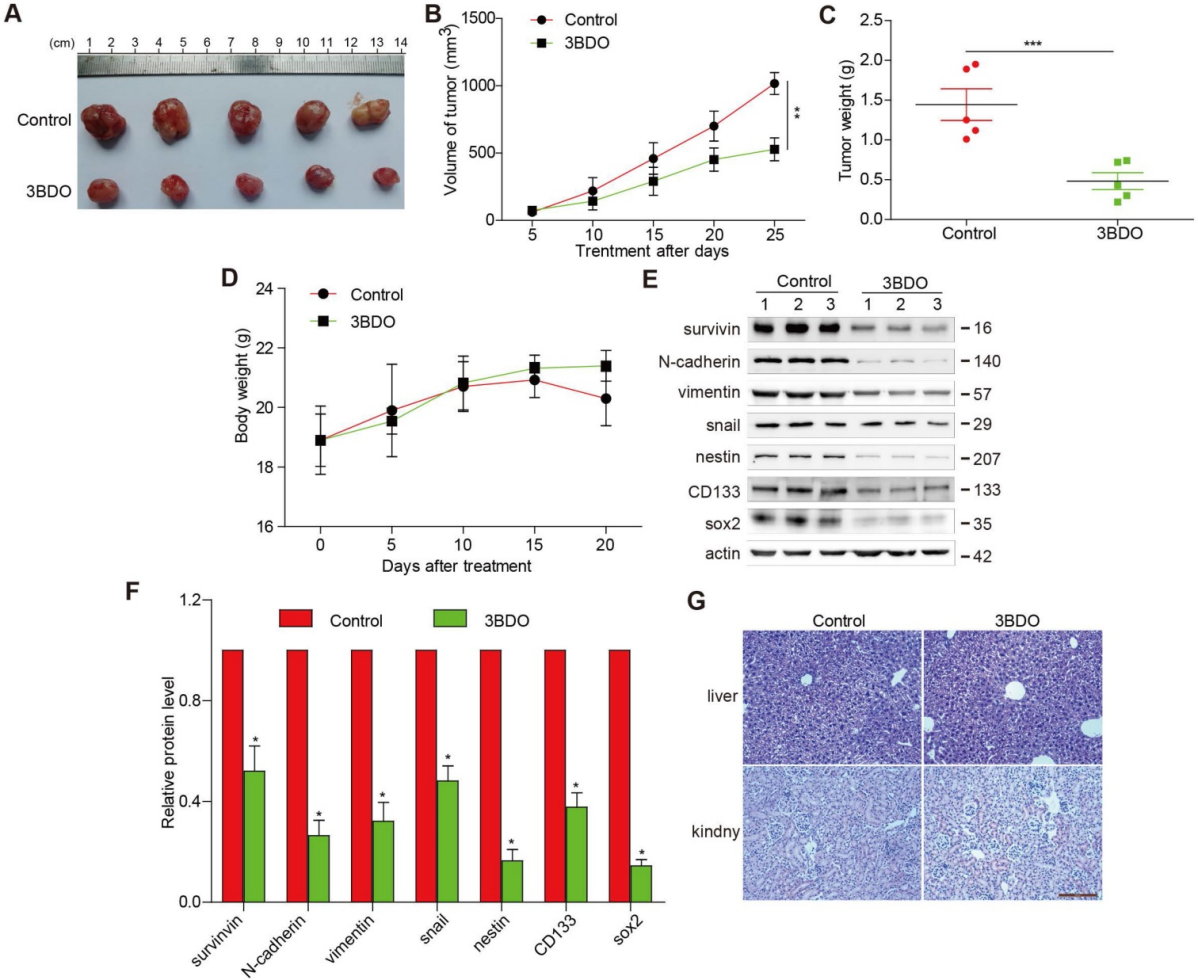
** 3BDO decreases glioma growth in a xenograft mouse model.** (A) The decrease in the size of xenograft U87 tumors was recorded using photographs (Bar,1 cm). The tumor volume (B), tumor weight (C), and bodyweight of the mice (D) were measured every 5 days. (E-F) At the end of the experiments, the tumor tissues were excised from the mice, and western blotting analysis was used to detect the protein expression levels, and the gels were analyzed and (G) the kidney and liver tissues were obtained and stained (Bar, 500 μm). n=3 or n=4 in each group. All experiments were performed in triplicate. *, P < 0.05; **, P < 0.01; ***, P < 0.001, compared with the control (0 μM).

3BDO treatment (80 mg/kg/day) over a period of 25 days resulted in significantly lower tumor weight and volume (Figure 5 A-C), without affecting the bodyweight of the mice (Figure 5D), compared with the untreated groups. Additionally, we confirmed that the expression of endogenous survivin, snail, N-cadherin, vimentin, and GSC markers, such as nestin, sox2, and CD133 in tumors dissected from the U87 xenograft mice were suppressed by 3BDO (Figure 5E-F). These findings are in agreement with the *in vitro* findings, suggesting that 3BDO suppresses tumor growth by inhibiting survivin-mediated EMT and stemness in glioblastoma xenograft models, thereby highlighting its potential as a therapeutic GBM candidate. Moreover, in order to detect the liver and kidney toxicity of the drug, when the end of the experiment was reached, we took the kidney and liver of the mouse and stained the HE section. It was found that the liver and kidney of the mice in the control group and the 3BDO group did not occur damage (Figure 5G).

## Discussion

Despite the advent of curative GBM therapy that involves the combination of surgical resection and chemoradiotherapy, GBM patient prognosis is still abysmal due to its high recurrence and metastatic ability. Therefore, novel treatment agents need to be urgently investigated. This study presents 3BDO, a known mTOR activator, as a likely EMT suppressor in GSC cells that exerts its effects through survivin downregulation.

3-benzyl-5-((2-nitrophenoxy) methyl)-dihydrofuran-2(3H)-one (3BDO), a novel mTOR activator, has demonstrated a variety of biological activity in addition to autophagy suppression 25, 26. It has been reported that 3BDO can improve cognitive defects by regulating autophagy in APP/PS1 AD mice models 19. In addition, 3BDO may be beneficial for the treatment of cardiovascular diseases through autophagy suppression 27. Moreover, 3BDO could inhibit the production of inflammatory cytokines both *in vivo* and *in vitro* 22. However, the effects of 3BDO in cancer have seldom been reported. Zhao et al. showed that 3BDO in combination with heat shock protein 90 (HSP90) inhibitor 4-(3-(7-(diethylamino)-2-oxo-2H-chromen-3-yl)-5-phenyl-4,5-dihydro-1H-pyrazol-1-yl) benzoic acid (DPB) could promote the effectiveness of A549 cell apoptosis 23. This study explored for the first time the inhibitory effects of 3BDO on GBM and GCS cells.

EMT has been proven to be closely involved in tumor development. It exerts its effects by maintaining tumor stemness, enhancing drug resistance, and enhancing cellular invasiveness - all of which greatly enhance tumor growth 28. GSCs are infamous for their high levels of resistance towards chemoradiotherapy and their potent ability to initiate tumor growth 6, 8. Therefore, it is essential to identify novel methods of treatment that can inhibit EMT and GSCs for the treatment of GBM. Several agents have been identified as EMT suppressors and are able to inhibit GSCs. In this study, we proved that 3BDO exerted anti-invasive and anti-migratory properties, which may reverse EMT progression in GBM. Moreover, we demonstrated that 3BDO inhibited GSC cell growth by suppressing stemness.

Survivin has been shown to be significantly upregulated in GBM and may play a role in promoting GBM development. GBM patients with higher survivin expression had shorter survival times compared with patients with lower survivin expression. In addition, EMT regulation has been reported to be mediated by survivin in GBM.

Liu et al. reported that IGF-1 triggered EMT in hepatocellular carcinoma by activating survivin 29. Additionally, Lee et al. showed that TGF-β regulated the EMT process by upregulating survivin 30. It has also been reported that the silencing of survivin expression could suppress EMT activation to decrease the invasive and migratory abilities of HCC cells 31. Knockdown of survivin results in inhibition of epithelial to mesenchymal transition in retinal pigment epithelial cells. Furthermore, Guvenc et al. reported that the suppression of survivin could impair GSC survival 18. Moreover, brexpiprazole has been shown to promote GSC sensitivity to chemotherapy drugs by downregulating survivin 32. Therefore, targeting survivin may be a promising strategy for the management of GBM. 3BDO, an autophagy inhibitor, was recently shown to be able to regulate TGFB2 in a manner that does not activate autophagy 22. In our study, we found that 3BDO could inhibit GBM cells and GSCs via survivin downregulation. Moreover, GBM cells and GSCs overexpressing survivin appeared to develop 3BDO resistance. This suggests that survivin may be a target of 3BDO. However, how 3BDO down-regulates survivin requires further exploration. In addition, it has been reported that activating or inhibiting autophagy by inhibiting or activating the mTOR pathway can inhibit the growth of gliomas by breaking the balance of autophagy. In our research, we found that 3BDO may inhibit the growth of gliomas by acting on survivin. Whether 3BDO inhibits the growth of gliomas by additionally inhibiting autophagy also requires further follow-up investigations.

Most agents that exert an anti-GBM effect *in vitro* remain ineffective *in vivo*. Previous drug candidates that have been shown to inhibit tumor growth in a subcutaneous GBM model failed to suppress GBM in the CNS due to the existence of the blood-brain barrier (BBB). Additional experiments evaluating the ability of 3BDO to inhibit glioma in a mouse stereotactic brain model are needed. Moreover, the safety and efficacy profiles of these drug candidates must be considered, especially for the development of anti-cancer agents. In previous investigations, 3BDO has been used in *in vivo* experiments, including experiments involving the CNS, and showed a good safety profile 26, 33. In our study, we found that 3BDO suppressed GBM growth in a subcutaneous GBM model in a manner similar to that demonstrated in our *in vitro* experiments. The lack of significant changes in overall mice body weight as well as the kidney and liver histopathology confirmed that 3BDO was favorably tolerated.

Overall, we proved that 3BDO successfully suppressed EMT and stemness in GBM both *in vivo* and *in vitro.* Additionally, we identified survivin as a potential target of 3BDO. We also demonstrated, for the first time, that 3BDO exerted anti-GBM properties using a subcutaneous glioma model, which was consistent with its mechanism of action demonstrated in the *in vitro* experiments. Therefore, 3BDO is a potential therapeutic drug for the treatment of GBM.
